# Cancer Cells’ Metabolism Dynamics in Renal Cell Carcinoma Patients’ Outcome: Influence of GLUT-1-Related hsa-miR-144 and hsa-miR-186

**DOI:** 10.3390/cancers13071733

**Published:** 2021-04-06

**Authors:** Mariana Morais, Francisca Dias, Inês Nogueira, Anabela Leão, Nuno Gonçalves, Luís Araújo, Sara Granja, Fátima Baltazar, Ana L Teixeira, Rui Medeiros

**Affiliations:** 1 Molecular Oncology and Viral Pathology Group, IPO-Porto Research Center (CI-IPOP), Portuguese Oncology Institute of Porto (IPO-Porto), Research Center-LAB2, E Bdg 1st Floor, Rua Dr António Bernardino de Almeida, 4200-072 Porto, Portugal; mariana.gomes.morais@ipoporto.min-saude.pt (M.M.); francisca.carvalho.dias@ipoporto.min-saude.pt (F.D.); inescmnogueira@gmail.com (I.N.); ruimedei@ipoporto.min-saude.pt (R.M.); 2 ICBAS, Abel Salazar Institute for the Biomedical Sciences, University of Porto, Rua Jorge Viterbo Ferreira 228, 4050-513 Porto, Portugal; 3 Research Department of the Portuguese League against Cancer Regional Nucleus of the North (LPCC—NRNorte), Estrada da Circunvalação 6657, 4200-177 Porto, Portugal; 4 Clinical Chemistry Department, Portuguese Oncology Institute of Porto (IPO-Porto), Rua Dr António Bernardino de Almeida, 4200-072 Porto, Portugal; anabelapereiraleao@gmail.com (A.L.); nuno.d.goncalves@gmail.com (N.G.); larauj@gmail.com (L.A.); 5 Life and Health Sciences Research Institute (ICVS), School of Medicine, Campos de Gualtar, University of Minho, 4710-057 Braga, Portugal; saragranja@med.uminho.pt (S.G.); fbaltazar@med.uminho.pt (F.B.); 6 ICVS/3B’s—PT Government Associate Laboratory, 4710-057 Braga, Portugal; 7 ICVS/3B’s—PT Government Associate Laboratory, 4835-258 Guimarães, Portugal; 8 Biomedical Reasearch Center (CEBIMED, Faculty of Health Sciences, Fernando Pessoa University (UFP), Praça 9 de Abril 349, 4249-004 Porto, Portugal; 9 Faculty of Medicine (FMUP), University of Porto, 4200-319 Porto, Portugal

**Keywords:** renal cell carcinoma, microRNAs, biomarkers, Warburg effect, glucose transporter 1

## Abstract

**Simple Summary:**

Renal cell carcinoma (RCC) is a metabolic associated cancer and the most common and lethal neoplasia in the adult kidney. This study aimed to understand the potential role of hsa-miR-144-5p and hsa-miR-186-3p (which target Glucose Transporter 1—GLUT-1) in clear cell RCC (ccRCC) glycolysis status, as well as their potential as biomarkers. A decrease of intracellular levels of these miRNAs and increase of their excretion was associated with an increase of GLUT-1’s levels and glycolysis’ markers. RCC patients presented higher plasmatic levels of hsa-miR-186-3p than healthy individuals and hsa-miR144-5p’s higher levels were associated with early clinical stages of RCC. Additionally, patients with low plasmatic levels of hsa-miR-144-5p and high plasmatic levels of hsa-miR-186-3p (high-risk group) showed a worse overall survival. Overall, these results indicate that circulating hsa-miR-144-5p and hsa-miR-186-3p may be potential biomarkers of ccRCC prognosis.

**Abstract:**

The cancer cells’ metabolism is altered due to deregulation of key proteins, including glucose transporter 1 (GLUT-1), whose mRNA levels are influenced by microRNAs (miRNAs). Renal cell carcinoma (RCC) is the most common and lethal neoplasia in the adult kidney, mostly due to the lack of accurate diagnosis and follow-up biomarkers. Being a metabolic associated cancer, this study aimed to understand the hsa-miR-144-5p and hsa-miR-186-3p’s potential as biomarkers of clear cell RCC (ccRCC), establishing their role in its glycolysis status. Using three ccRCC lines, the intra- and extracellular levels of both miRNAs, GLUT-1’s mRNA expression and protein levels were assessed. Glucose consumption and lactate production were evaluated as glycolysis markers. A decrease of intracellular levels of these miRNAs and increase of their excretion was observed, associated with an increase of GLUT-1’s levels and glycolysis’ markers. Through a liquid biopsy approach, we found that RCC patients present higher plasmatic levels of hsa-miR-186-3p than healthy individuals. The Hsa-miR144-5p’s higher levels were associated with early clinical stages. When patients were stratified according to miRNAs plasmatic levels, low plasmatic levels of hsa-miR-144-5p and high plasmatic levels of hsa-miR-186-3p (high-risk group) showed the worst overall survival. Thus, circulating levels of these miRNAs may be potential biomarkers of ccRCC prognosis.

## 1. Introduction

From all the adult kidney cancers, renal cell carcinoma (RCC) accounts for 80% of the cases, being the most common and the most lethal urological cancer due to its high metastatic potential [1]. There are several subtypes of RCC that differ not only histologically but genetically, molecularly and clinically [2]. Among them, the most common, approximately 80%, and lethal one is the clear cell RCC (ccRCC) [3]. Due to ccRCC location and the absence of a standard screening test, one third of RCC patients are diagnosed with local invasive or metastatic disease and 20–40% of patients submitted to nephrectomy will present local recurrence or distant metastasis [4]. Moreover, treatment options in this tumor stage are limited and are associated with high rates of therapy resistance [5]. Thus, there is an urgent need for accurate biomarkers for diagnosis, prognosis and therapeutic response.

Among the several types of biomarkers studied in RCC are microRNAs (miRNAs), a class of short non-coding RNAs that negatively regulate gene expression at a post-transcriptional level through the binding to complementary sequences in the 3′ untranslated region (3′ UTR) of messenger ribonucleic acids (mRNAs) [6]. These non-coding RNAs are secreted in several body fluids (such as serum, plasma, saliva and urine) and their circulating levels have been associated with cancer development and clinical outcomes in different tumor models [7]. In ccRCC, there are several studies showing the association between circulating miRNA profiles and histology, staging and clinical endpoints, such as patients’ survival and therapy response [8,9,10,11,12].

The loss or inactivation of the von Hippel Lindau (VHL) gene is a key feature associated with ccRCC development that leads to an increase of Hypoxia Inducible Factor alpha (HIF-α) and triggers a hypoxic response from the cell, even in normoxic conditions [13]. One of the genes whose transcription is induced is Glucose Transporter 1 (GLUT-1), a transporter responsible for the passive entry of glucose into the cell [14]. GLUT-1’s upregulation in cancer is one of the main drivers of the Warburg effect, also known as aerobic glycolysis, which states the preference of cancer cells to use glycolysis instead of oxidative phosphorylation, even in the presence of oxygen [15,16]. Thus, GLUT-1’s expression deregulation is directly related with the disruption of cellular metabolism, which is one of the cancer hallmarks proposed by Hanahan and Weinberg in 2011 [17].

GLUT-1 has been shown to be directly regulated by hsa-miR-144-5p and hsa-miR-186-3p, whose expression deregulation has already been observed in several tumor models [18,19,20,21]. Since ccRCC is considered a metabolic associated cancer, the aim of this study was to understand the potential of hsa-miR-144-5p and hsa-miR-186-3p as biomarkers of ccRCC by establishing their role in the glycolysis status of ccRCC.

## 2. Results

### 2.1. Overexpression of GLUT-1 in ccRCC and Its Association with an Aerobic Glycolysis Shift

**Figure 1:**
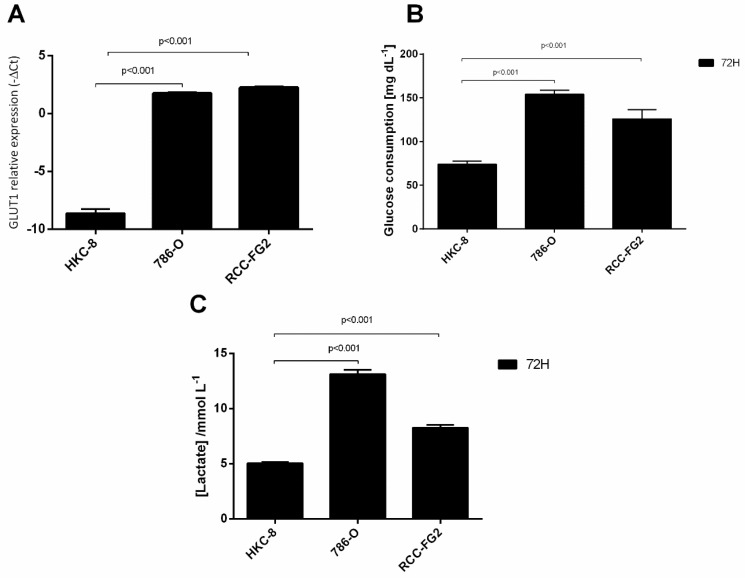
Variation of GLUT-1, glucose consumption and lactate production in HKC-8, 786-O and RCC-FG2 cell lines. (**A**) Variation of the levels of GLUT-1 mRNA in HKC-8, 786-O and RCC-FG2 (mean ± SE); (**B**) Variation of glucose consumption in HKC-8, 786-O and RCC-FG2 at the end of 72 h (mean ± SD); (**C**) Variation of lactate production in HKC-8, 786-O and RCC-FG2 at the end of 72 h (mean ± SD).

To understand the glycolysis state of ccRCC, we performed an in vitro study in which we evaluated the GLUT-1 mRNA levels, GLUT-1 protein levels, glucose consumption and lactate production in HKC-8, 786-O and RCC-FG2 cell lines, that mimic the ccRCC progression. According to our results, GLUT-1 mRNA levels were significantly higher in 786-O (fold increase = 1360) and RCC-FG2 cell lines (fold increase = 1910) when compared with HKC-8 cell line (Figure 1A). As expected, the glucose consumption was significantly higher in 786-O (2.1 times) and in RCC-FG2 (1.7 times) when comparing with HKC-8 (Figure 1B). The same happened with lactate concentration in the medium that was 2.6 and 1.6 times higher in 786-O and RCC-FG2, respectively (Figure 1C).

### 2.2. Intra- and Extracellular Levels of hsa-miR-144-5p and hsa-miR-186-3p Deregulation in ccRCC Cell Lines

**Figure 2:**
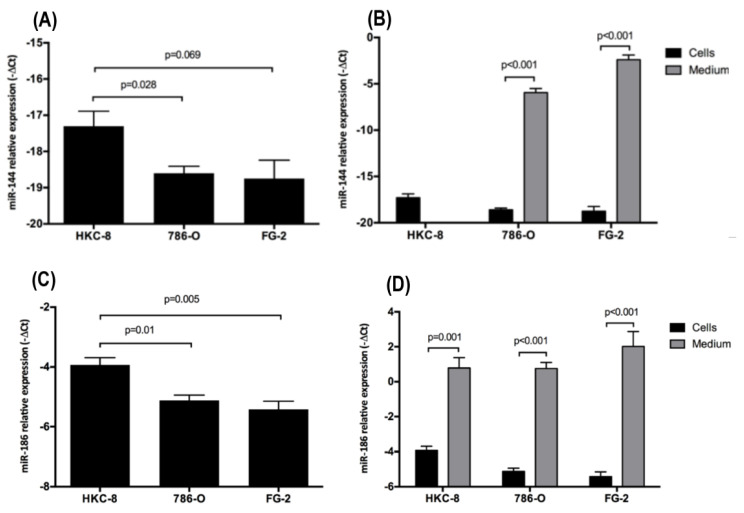
Variation of the intracellular and extracellular levels of hsa-miR-144-5p and hsa-miR-186-3p in HKC-8, 786-O and RCC-FG2 cell lines. (**A**) hsa-miR-144-5p levels in HKC-8, 786-O and RCC-FG2 cell lines; (**B**) hsa-miR-144-5p levels in HKC-8, 786-O and RCC-FG2 medium; (**C**) hsa-miR-186-3p levels in HKC-8, 786-O and RCC-FG2 cell lines; (**D**) hsa-miR-186-3p levels in HKC-8, 786-O and RCC-FG2 medium. The bars represent the -ΔCT of miRNAs expression normalized to RNU44 (mean ± SE).

Since GLUT-1’s mRNA is directly targeted by hsa-miRNA-144-5p and hsa-miRNA-186-3p [18,19,20,21], we performed an in vitro study to evaluate the intra- and extracellular levels of both miRNAs in HKC-8, 786-O and RCC-FG2 cell lines. According to our results, the intracellular levels of hsa-miR-144-5p are significantly reduced (fold change = 0.41) in 786-O and RCC-FG2 (fold change = 0.37) when compared with HKC-8 (Figure 2A). Regarding hsa-miR-186-3p, we also observed a significant reduction in its intracellular levels in 786-O (fold change = 0.44) and RCC-FG2 (fold change = 0.36) when compared with HKC-8 (Figure 2C). On the other hand, we did not detect the hsa-miR-144-5p in HKC-8 cell media, but it was detected in high amounts in 786-O and RCC-FG2 cell media. In fact, hsa-miR-144-5p levels were significantly higher in the medium when compared with the intracellular ones in both cell lines, with a fold increase of 64 472 in 786-O and 83 529 in RCC-FG2 (Figure 2B). Regarding the hsa-miR-186-3p, in the three cell lines the extracellular levels were higher than the intracellular ones, with a 26-fold increase in HKC-8, a 59-fold increase in 786-O and a 174-fold increase in RCC-FG2 (Figure 2D).

### 2.3. Glucose Interference in hsa-miR-144-5p, hsa-miR-186-3p, GLUT-1 mRNA and Protein Expression Patterns

**Figure 3:**
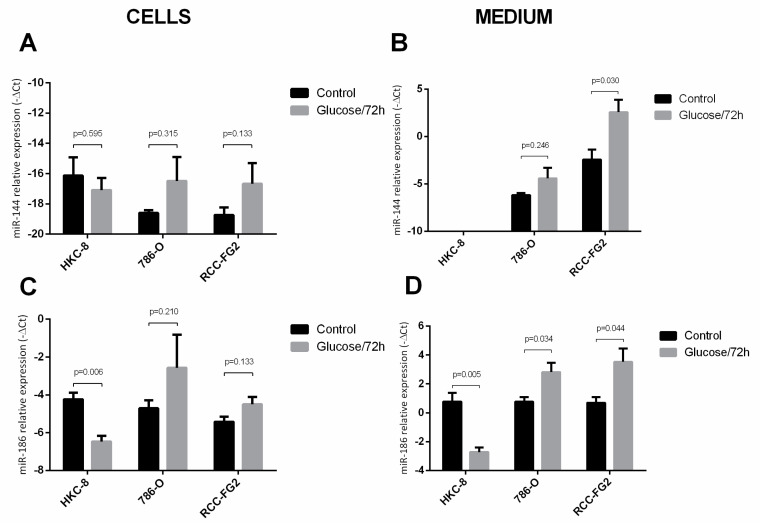
Variation of the intracellular and extracellular levels of hsa-miR-144-5p and hsa-miR-186-3p in HKC-8, 786-O and RCC-FG2 after a 72 h stimulus with glucose. (**A**) hsa-miR-144-5p levels in HKC-8, 786-O and RCC-FG2 cell lines with and without the glucose stimulus; (**B**) hsa-miR-144-5p levels in HKC-8, 786-O and RCC-FG2 culture medium with and without glucose stimulus; (**C**) hsa-miR-186-3p levels in HKC-8, 786-O and RCC-FG2 cell lines with and without the glucose stimulus; (**D**) hsa-miR-144-5p levels in HKC-8, 786-O and RCC-FG2 culture medium with and without glucose stimulus; The bars represent the -ΔCT of miRNAs expression normalized to RNU44. (mean ± SE).

To understand the effect of one glucose stimulus in the expression of hsa-miR-144-5p and hsa-miR-186-3p, we applied a 25mM D-Glucose stimulus to the cell lines and then quantified the cells miRNAs levels after 72 h. Regarding hsa-miR-144-5p, we did not observe any differences among the intracellular levels with, or without, glucose stimulus in any of the three cell lines (Figure 3A). However, we observed a significant increase in the extracellular levels of this miRNA in RCC-FG2 cell line (fold increase = 31.8), which could suggest an increase of miRNAs secretion (Figure 3B). Regarding the hsa-miR-186-3p’s intracellular levels, we only observed a decrease in HKC-8 cell line (fold change = 0.09) (Figure 3C). Concerning the extracellular levels of this miRNA, we observed a significant decrease in HKC-8 (fold change = 0.21) and a significant increase in both 786-O (fold increase = 4.2) and RCC-FG2 (fold increase = 7.11) (Figure 3D).

**Figure 4:**
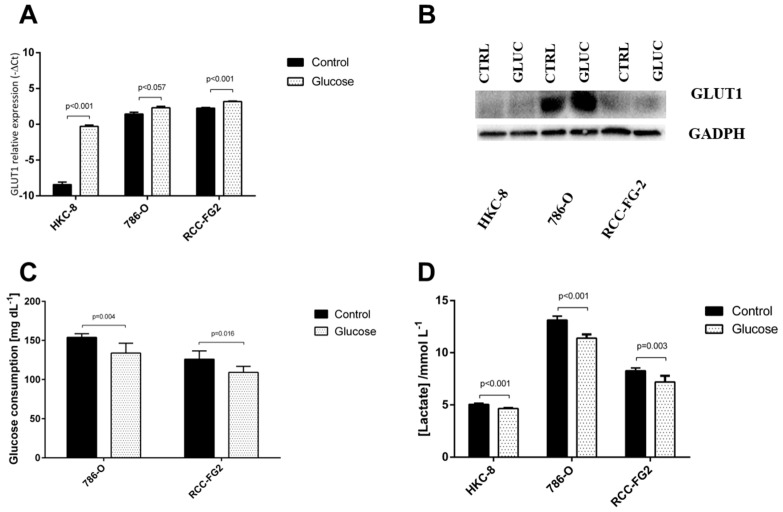
Variation of GLUT-1 and glucose consumption and lactate production in HKC-8, 786-O and RCC-FG2 after a 72 h stimulus with glucose. (**A**) Variation of the relative expression levels of GLUT-1 mRNA in HKC-8, 786-O and RCC-FG2 with and without glucose stimulus (mean ± SE); (**B**) protein levels in the three cell lines according to Western blot analysis with and without glucose stimulus; (**C**) variation of glucose consumption in HKC-8, 786-O and RCC-FG2 with and without glucose stimulus (mean ± SD); (**D**) variation of lactate production in HKC-8, 786-O and RCC-FG2 with and without glucose stimulus (mean ± SD).

Similarly, we also evaluated the impact of the glucose stimulus in glycolysis by analyzing GLUT-1 mRNA and protein levels, the amount of glucose consumed and lactate concentration in the medium. There was a significant increase in GLUT-1 mRNA levels in HKC-8 (fold increase = 282) and RCC-FG2 (fold increase = 1.9) (Figure 4A). The Western blot showed an increase of GLUT-1 protein expression in the tumoral cell lines when compared with the normal one, but following the glucose stimulus, there was no significant difference in GLUT-1 protein expression (Figure 4B). Nevertheless, this stimulus led to a significant reduction of glucose consumption in 786-O (13%) and RCC-FG2 (13%) and of lactate concentration in the medium in the three cell lines (HKC-8 showed a reduction of 8%; 786-O showed a reduction of 13%, and RCC-FG2 showed a reduction of 13%) (Figure 4C,D).

### 2.4. hsa-miR-144-5p and hsa-miR-186-3p Plasma Levels in RCC Patients and Their Association with Clinicopathological Characteristics

**Figure 5:**
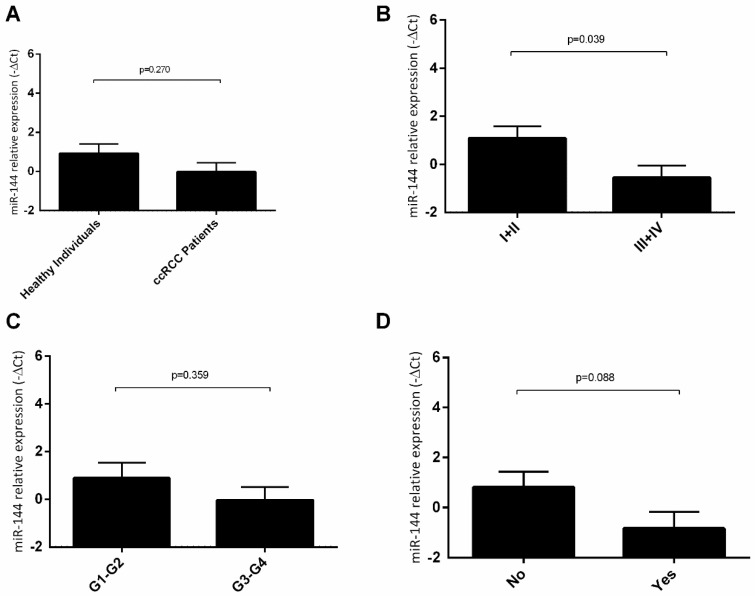
Variation of plasmatic levels of hsa-miR-144-5p according to clinicopathological characteristics (Mean ± SE); (**A**) healthy individuals vs. ccRCC patients; (**B**) clinical stage I + II vs. clinical stage III + IV; (**C**) Fuhrman grade G1-G2 vs. Fuhrman grade G3-G4; (**D**) no recurrence vs. recurrence.

According to our results, there were no statistically significant differences in the plasma levels of hsa-miR-144-5p in ccRCC patients, when compared to healthy individuals (Figure 5A). Regarding the clinicopathologic characteristics, we observed that RCC patients with I−II tumor stages presented a significantly higher level of hsa-miR-144-5p when compared with patients with III−IV tumor stages (fold decrease = 3.12) (Figure 5B). However, we did not observe any statistically significant differences among the miRNA levels and Furhman grade (Figure 5C) or recurrence status (Figure 5D).

**Figure 6:**
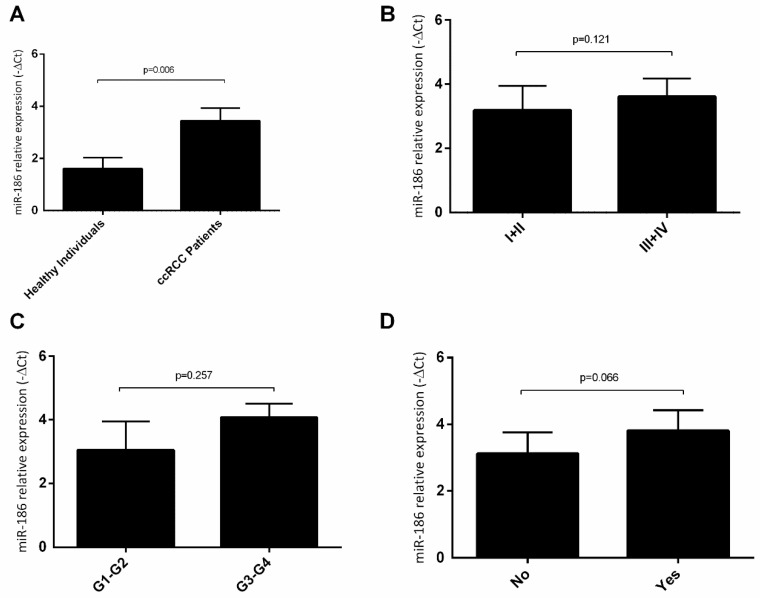
Variation of plasmatic levels of hsa-miR-186-3p according to clinicopathological characteristics (mean ± SE); (**A**) healthy individuals vs. ccRCC patients; (**B**) clinical stage I + II vs. clinical stage III + IV; (**C**) Fuhrman grade G1-G2 vs. Fuhrman grade G3-G4; (**D**) no recurrence vs. recurrence.

Regarding hsa-miR-186-3p, we observed that RCC patients presented higher plasma levels of this miRNA, when compared to healthy individuals (fold increase = 3.58) (Figure 6A). However, we did not observe any statistically significant differences associated with clinical stage (Figure 6B), Furhman grade (Figure 6C) neither with recurrence (Figure 6D).

The patients of this cohort were stratified according to their low or high plasma levels of hsa-miR-144-5p and hsa-miR-186-3p. To define low and high expression of each miRNA, the mean value of -ΔCt for each miRNA was considered. Then, three groups were established considering the combination of hsa-miR-144-5p and hsa-miR-186-3p high plasma levels and hsa-miR-144-5p and hsa-miR-186-3p low plasma levels which allowed the definition of high, intermediate and low risk groups. The high risk group was constituted of patients combining low expression of hsa-miR-144-5p and high expression of hsa-miR-186-3p. The intermediate risk group combined both patients with high hsa-miR-144-5p and high hsa-miR-186-3p expression and patients with low hsa-miR-144-5p and low hsa-miR-186-3p expression. The low-risk group combined patients with a high expression of hsa-miR-144-5p and low expression of hsa-miR-186-3p.

**Figure 7:**
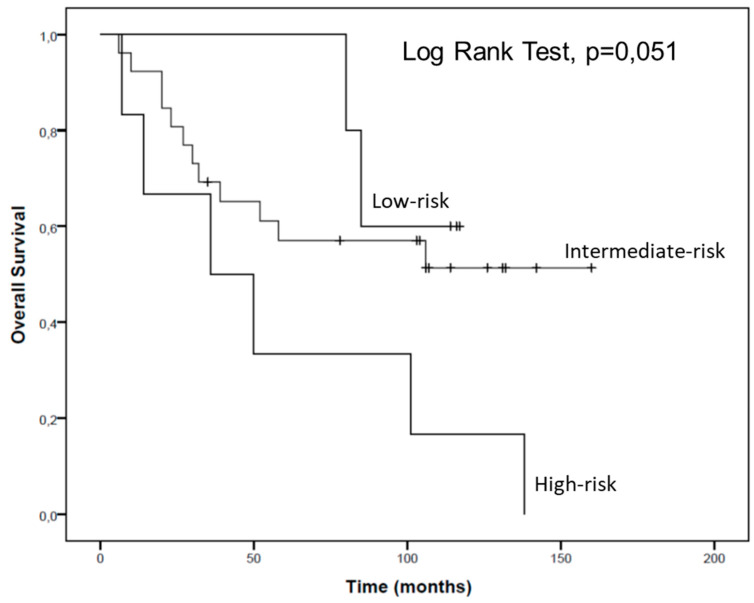
Overall survival of RCC patients according to hsa-miR-144-5p and hsa-miR-186-3p plasma expression levels.

According to our results, patients from the high-risk group present a tendential lower overall survival than patients from the intermediate and low-risk groups (57.7 vs. 100.7 vs. 103.2 months, respectively) (Figure 7).

## 3. Discussion

Since 2011, the deregulation of cellular metabolism has been considered one hallmark of cancer, however this cellular alteration was studied long before that [14]. Despite having been first described in the 1920s, and having already been observed in several tumor models, the selective advantage of the Warburg effect is not yet fully clarified [15]. This effect highly depends on the disruption of several signaling pathways, such as the deregulation of metabolic enzymes and transport systems expression, namely the GLUT-1 transporter [22]. In our study, we have observed an increase of both GLUT-1’s mRNA and protein levels as well as an increase of glucose consumption and lactate production in the tumoral cells. These results support the occurrence of the Warburg effect in ccRCC, which had been previously reported by other authors [16]. By switching to anaerobic glycolysis, cancer cells metabolize glucose into pyruvate and then lactate, instead of carbon dioxide (oxidative phosphorylation), leading to an increase of lactate production. Like this, they do not need oxygen, but they also produce less energy. To make up for the less energy obtained in the deviation of glucose metabolism into aerobic glycolysis, the cells need to increase glucose consumption, which is achieved through the increase of both GLUT-1’s mRNA and protein levels and also of the metabolic capacity. In fact, in RCC, the upregulation of GLUT-1 is already reported, since GLUT-1 transcription is known to be induced by HIF-α, a key transcription factor involved in this tumor biology [13]. Because of these properties, RCC has been considered a good model of a cancer caused by a metabolic disruption [23,24].

Nevertheless, the increase of GLUT-1’s mRNA levels can also be potentiated by the deregulation of several miRNAs. Both miRNAs selected for the present study have been implicated in binding to the 3′-UTR of GLUT-1’s mRNA and, consequently inhibiting its translation into protein [18,19]. In our study, we have observed a decrease in the intracellular levels of both hsa-miR-144-5p and hsa-miR-186-3p in the ccRCC cell lines and extracellularly, hsa-miR-144-5p was only present in the culture medium of the cancer cell lines. We have also observed a significant difference between the intra- and extracellular levels of both these miRNAs in cancer cell lines, with a higher expression of both miRNAs in the culture medium. These results may suggest a suppression of these miRNAs’ production followed by an increase in their excretion, as an additional effort of cancer cells to increase GLUT-1’s mRNA expression and better use aerobic glycolysis as their preferential metabolic mechanism. In fact, the overexpression of miR-186, through agomiR-186 transfection, was able to reverse the Warburg effect in gastric cancer cell lines, decreasing its glucose consumption and lactate production, consequently inhibiting cell proliferation and increasing apoptosis [25]. In the same way, miR-144’s overexpression, using a miR-144 mimic, was able to reverse the Warburg effect in a lung cancer cell line, reducing its glucose uptake and lactate production [18]. Thus, we suggest that the decrease of these miRNAs’ levels in the cell, through their secretion to the microenvironment, may play a major role in Warburg effect promotion in RCC. 

Even though these miRNAs were already reported as deregulated in ccRCC [26,27,28], this is the first study assessing the secretion levels of these miRNAs in ccRCC cell lines. There are two main mechanisms of miRNAs secretion to the microenvironment: incorporated into protein complexes or inside extracellular vesicles (EVs). While protein-bound miRNAs are apparently nonspecific remnants resulting from cellular activity or cell death, EVs have been gaining attention from the scientific community, since the exchange of vesicles between cells will have an impact on the recipient cell phenotype, playing a major role in the tumor microenvironment dynamics [29]. In fact, Dias and collaborators have recently shown that EV-miRNAs could be potential biomarkers of metastatic ccRCC and their enrichment in the presence of the tumor may be an epigenetic mechanism to support tumor development [12]. Therefore, the secretion of miR-186 and miR-144 by EVs can both increase GLUT-1’s expression of the donor cells to promote Warburg effect but also inhibit GLUT-1’s expression of the recipient cells in a competitive attempt for the glucose available. In the future, it would be interesting to verify this hypothesis to further clarify the microenvironment dynamics.

Interestingly, when we administrated an acute glucose stimulus to the three cell lines culture mediums, there was a reduction of hsa-miR-186-3p production and consequent excretion in HKC-8 cells and an increase of hsa-miR-186-3p and hsa-miR-144-5p excretion in the two ccRCC cell lines. This increase of hsa-miR-144-5p and hsa-miR-186 excretion in ccRCC and the decrease in the production of hsa-miR-186-3p by HKC-8 was always followed by an increase of GLUT-1 mRNA levels, showing the importance of these miRNAs for the regulation of GLUT-1’s mRNA levels. On the other hand, we observed no alteration in GLUT-1’s protein levels and a decrease of both glucose consumption and lactate production. This may be explained by the existence of additional miRNAs that bind incompletely to GLUT-1 mRNA, not degrading it but only inhibiting its translation into protein. In fact, there are other miRNAs, such as miR-495, miR-132, miR-340 and miR-22, that have been proved to target GLUT-1 but have not yet been studied in ccRCC [16]. Thus, additional studies should be performed to understand if they are responsible for the inhibition of GLUT-1 mRNA translation. Nevertheless, these results, either in normal or in cancer cell lines, also highlight the modulation capacity of external stimulus, like alterations in the levels of glucose intake, in the cellular microenvironment and in their epigenetic profile. In fact, miR-144 has been previously shown to be upregulated in the blood and blood fractions of type 2 diabetics, showing a relation between high glucose environments and increased levels of miR-144 in circulation [30,31]. Additionally, recent studies have shown that EV levels were significantly raised in individuals with type 2 diabetes or obesity, which may indicate that EVs are in fact the mechanism behind these miRNA secretions [32,33]. Thus, we hypothesize that, in response to higher glucose levels in the medium, there is an increased production of EVs, and a consequent increase in miRNA secretion.

Because of these promising in vitro results, we have studied both miRNA-144 and miRNA-186-3p plasmatic levels in a cohort of ccRCC patients and healthy individuals. To the best of our knowledge, our study is the first one to evaluate the plasma levels of hsa-miR-144-5p and of hsa-miR-186-3p. In our study we have observed a decrease of hsa-miR-144-5p’s plasmatic levels in RCC patients with more advanced clinical stage tumors. The downregulation of hsa-miR-144-5p in RCC patients with poorer prognostics had already been shown by Yamada and coworkers, who analyzed the expression levels of this miRNA in tissue specimen of RCC patients [34]. Besides GLUT-1, according to miRTarBase (version 8.0), hsa-miR-144-5p directly targets other oncogenes such as RUNX-1, TGIF1, ROCK1, ROCK2, MET, CCNE1, SMAD4 [35]. Thus, the decrease of hsa-miR-144-5p production or its augmented inhibition will lead to the overexpression of these genes and will favor a worse prognosis. The association of low miR-144-5p levels in both tissues and plasma samples of ccRCC patients with a poorer prognosis observed by Yamada and coworkers and in this present study, indicates the biomarker potential of this miRNA in a future liquid biopsy approach.

In our study, we have observed an increase of hsa-miR-186-3p plasmatic levels in RCC patients when compared with healthy individuals. Jiao and coworkers previously described a downregulation in RCC tissues when compared with the normal adjacent tissues, but they have not studied the levels of this miRNA in the plasma samples of the patients and they do not compare it with healthy individuals’ tissues [28]. Our results could be explained by an increased excretion of this miRNA by tumor cells which would increase its plasmatic levels and at the same time would lead to decreased levels in cancer tissues. On the other hand, the dual role of hsa-miR-186-3p has already been highlighted. Even though it directly targets oncogenes such as GLUT-1, which are studied in this paper, it has been shown to directly target tumor suppressor genes such as caspase 10 and epiregulin (inhibiting cell apoptosis and increasing cell proliferation), RETREG 1 (inducing G1-S transition and migration), APAF-1 (inducing invasion), PPM1B (promoting G1-S transition and proliferation) and SENP1 (promoting NF-kB pathway and inducing tumor invasion) [36]. This dual role may explain the increased plasma levels of hsa-miR-186 in RCC patients observed in our study, but it also highlights the need of miRNAs profiles, that include several miRNAs in order to increase the sensibility and accuracy of these potential biomarkers and their introduction in clinic.

Moreover, it is important to highlight that, when we separated RCC patients in three risk groups according to their hsa-miR-144-5p and hsa-miR-186-3p plasmatic levels, we observed a tendential reduction in the overall survival in patients who belong to the high risk group, which show low plasmatic levels of hsa-miR-144-5p and high plasmatic levels of hsa-miR-186 (57.7 vs. 103.2 months).

Future research, with a larger cohort of ccRCC patients, is needed to validate the results reported. Moreover, the study of the mechanisms of these miRNA excretions, namely EVs, would be important to better understand the mechanisms behind their deregulation.

## 4. Materials and Methods 

### 4.1. Cell Lines and Cell Culture

In order to mimic the development of ccRCC, three renal cell lines were used: HKC-8, 786-O and RCC-FG2 [37]. The HKC-8 cell line is a human-derived normal proximal tubular epithelial renal cell line. The 786-O is a ccRCC cell line and RCC-FG2 is a metastatic ccRCC cell line. The two tumor cell lines do not express VHL. Both HKC-8 and RCC-FG2 were kindly provided by Dr. Klaas Kok from Groningen University (Netherlands) and 786-O cell line was kindly granted by Professor Cármen Jerónimo from IPO-Porto Research Center (Portugal).

Both 786-O and RCC-FG2 cells were kept in RPMI 1640 (1X) medium (Gibco ^®^, Gaithersburg, MD, USA), supplemented with 10% of FBS (Fetal Bovine Serum) (Gibco^®^, Gaithersburg, MD, USA) and 1% of Pen-Strep (Gibco). HKC-8 cells were maintained in DMEM/F12 medium (Gibco^®^, Gaithersburg, MD, USA), supplemented with ITS (Insulin-transferrin-selenium) (Sigma-Aldrich^®^, St. Louis, MO, USA), Pen-Strep (Gibco^®^, Gaithersburg, MD, USA), EGF (Epidermal Growth Factor) (Sigma-Aldrich^®^, St. Louis, MO, USA), Hepes buffer (Gibco^®^, Gaithersburg, MD, USA) and Hydrocortisone (Sigma-Aldrich^®^, St. Louis, MO, USA). The three cell lines were kept in an incubator with the following conditions: 37 °C temperature, 5% CO_2_ and humid atmosphere. Every 2 weeks in culture, all cells were tested for mycoplasma presence and were found to be free from contamination.

When cells had reached 80–90% confluence, the medium in which they were being maintained was collected for miRNA extraction and cells were trypsinized with trypsin-EDTA (1X) (Gibco^®^, Gaithersburg, MD, USA). Two million cells were counted using the EVE Automated Cell Counter (NanoEnTek, VWR^TM^, Radnor, Pennsylvania, USA), centrifuged to form a pellet for miRNA and mRNA extraction and the remaining cells were maintained in culture.

For each cell line, this procedure was repeated five times, both for the medium and for the cells.

### 4.2. Study Population and Sample Collection

miRNA-144 and miRNA-186’s potential as prognosis biomarker of RCC were analyzed through a hospital-based study. The 54 patients (age of 60.3 ± 12.1 years) with histopathologic diagnosis of RCC were recruited at the Portuguese Institute of Oncology of Porto from 1st September of 2003 to 30th July of 2013. From the 54 patients, 74.1% were males and 25.9% were females [8]. The clinical characteristics of each patient were obtained from their medical record. The extension of disease was classified accordingly to TNM classification system of the American Joint Committee on Cancer (AJCC) 2010, 7th edition. A further 53 healthy individuals (32.1% male and 67.9% female) with a mean age of 42.8 ± 16.1 years and no history of cancer, were randomly recruited from the north of Portugal and constituted the control group. This study was conducted according to the principles of the Helsinki Declaration and the local ethics committee at the Portuguese Institute of Oncology of Porto-Portugal has approved it (245/2013). Moreover, all the individuals signed a written informed consent in order to participate in the study. An 8 mL sample of peripheral blood was collected from all the individuals, through a standard method of intravenous collecting with Ethylenediamine tetra-acetic acid (EDTA) tubes. To separate the plasma fraction, the blood tubes were centrifuged for 5 min at 3000 rpm at room temperature. 

### 4.3. MiRNA and mRNA Isolation

Extraction of miRNA from the cells and the respective medium, as well as from the plasma samples, was performed using the GRS microRNA kit (Grisp Research Solutions^®^_,_ Porto, Portugal) with a protocol previously optimized in our lab [8]. Extraction of mRNA was performed with the GRS Total-Blood & Cultured Cells kit (Grisp Research Solutions^®^_,_ Porto, Portugal). We added an acid phenol-chloroform (5:1) solution (Thermo fisher^®^, Waltham, MA, USA) to the samples, which, after centrifugation at 15,000× *g* rpm at 5 °C for 15 min, allowed the separation of the RNA/microRNA phase. MicroRNA purification was performed using the GRS microRNA kit (Grisp Research Solutions^®^_,_ Porto, Portugal), with adjustments in the manufactured protocol. Concentration and purity of the isolated mRNA was then assessed by absorbance measurement at 260 and 280 nm using the NanoDrop Lite Spectrophotometer (Thermo fisher^®^, Waltham, MA, USA).

### 4.4. cDNA Synthesis

MiRNA samples served as templates for cDNA synthesis using a Taqman MicroRNA Reverse Transcription kit (Applied Biosystems^®^, Waltham, MA, USA) and sequence-specific stem-loop primers for hsa-miR-144-5p and hsa-miR-186, RNU44, RNU48 and RNU6b. mRNA samples served as templates for complementary DNA (cDNA) synthesis using a High Capacity cDNA Reverse Transcription Kit (Applied Biosystems^®^, Waltham, MA, USA). The thermal conditions for PCR amplification were optimized to 16 °C for 30 min, followed by 42 °C for 60 min and 85 °C for 10 min for miRNAs cDNA synthesis and 25 °C for 10 min, followed by 37 °C for 120 min and 85 °C for 5 min for mRNA.

### 4.5. Relative Quantification by Real-Time PCR

MiRNA and mRNA expression was assessed by quantitative real-time PCR using both StepOneTMqPCR Real-Time PCR machine and StepOnePlusTM qPCR Real-Time PCR machine. The reaction was performed using 1X Master mix (Applied Biosystems), with 1X probes (TaqMan microRNA Expression Assays, hsa-miR-144-5p: TM002148, hsa-miR-186: TM002285, RNU44: TM001094, RNU48: TM001006 and RNU6b: TM001093 or TaqMan mRNA Expression Assays, GLUT-1: Hs00892681_m, human GUSB (Beta Glucuronidase) endogenous control (Applied Biosystems^®^, Waltham, MA, USA) and cDNA sample. The behavior of RNU44, RNU48 and RNU6b expression was analyzed both in medium and inside the cell to choose the best endogenous control for miRNA normalization.

RNU44 presented more constant average Ct values and smaller standard deviations. Therefore, it was the endogenous control chosen to normalize the miRNA expression levels. GUSB was used as endogenous control to normalize mRNA results since its expression levels also remained constant [8,38].

The amplification conditions were as follows: holding stage 95 °C for 20 s, followed by 45 cycles of 95 °C for 1 s and 60 °C for 20 s. Three technical replicates were made for each sample.

For data analysis, StepOne Software v2.2 (Applied Biosystems^®^, Waltham, MA, USA) was used and the baseline and thresholds were set for each plate to create the threshold cycle (Ct) values for all the miRNAs and mRNAs in each sample. All quantifications were performed in duplicate and each plate had a negative control.

### 4.6. Lactate Production and Glucose Consumption Measurement

Some 180,000 cells were cultured, per well, in a 6-multi-well plate in the conditions previously described. In order to determine the basal glucose consumption and lactate production, the medium in which they were being maintained was collected and both lactate production and glucose consumption was measured recurring to GEM Premier 3000 (Instrumentation Laboratory) after 72 h.

To understand the effect of glucose stimulus on lactate production by the cells, 25 mM D-Glucose was added to each plate for a period of 72 h. Lactate production and glucose consumption was then measured using the same method. At this time point miRNA and mRNA were extracted and hsa-miR-144-5p, miR186, RNU44, RNU48, RNU6b, GLUT-1 and GUSB levels were quantified using the protocols described previously.

Both experiments were performed (six replicates for condition) in HKC-8, 786-O and RCC-FG2 cell lines.

### 4.7. Western Blotting

Cells were washed with PBS (pH 7.4), homogenized in lysis buffer (supplemented with protease inhibitors) for 20 min and then centrifuged at 1400× *g* for 15 min at 4 °C. The supernatants were then collected, and total protein was quantified by the Bradford Method (Bio-Rad Laboratories, Hercules, California, USA), using BSA as standard. Aliquots of 25 µg of total protein were separated on 10% polyacrylamide gel by SDS-PAGE and transferred onto a nitrocellulose membrane in 25 mM Tris-base/glycine buffer. Membranes were blocked with 5% milk in TBS/0.1% Tween (TBS-T; pH = 7.6) for 1 h at room temperature. After incubation overnight at 4 °C with the primary polyclonal antibody GLUT-1 (D3J3A, CellSignaling Technology, dilution 1:1000), membranes were washed in TBS/0.1% Tween and incubated with the secondary antibody coupled to horseradish peroxidase. Blots detection was done by chemiluminescence (Thermo Scientific Pierce ECL Western Blotting) using the ChemiDoc XRS + System (Bio-Rad Laboratories, Hercules, California, USA). GADPH (Santa Cruz Biotechnology, sc-32233, dilution 1:500) was used as loading control. A ChemiDoc system (Bio-Rad, Laboratories, Hercules, California, USA) was used for the detection and analysis of band intensity.

### 4.8. Statistical Analysis

Data analysis was performed by the computer software IBM SPSS Statistics for Windows (Version 24.0). Livak method (2^−∆∆CT^) and Student’s *T*-test or Wilcoxon rank-sum test (depending on whether samples assumed a normal distribution or not) were used to assess differences between the expression levels of the normalized miRNAs and mRNAs as well as differences in the metabolic capacity [39]. The differences between groups were considered significant when *p*-value < 0.05. Overall survival, determined by interval of time since the diagnosis until death or the last clinical visit, was accessed with the Kaplan−Meier method and log-rank test with all the samples.

## 5. Conclusions

The present study has shown the deregulation of metabolism in RCC and the presence of the Warburg effect in this tumor model. It has also revealed the potential of both hsa-miR-144-5p and hsa-miR-186-3p as future biomarkers of RCC. A future miRNA profile with biomarker potential of this tumor model could benefit from including these miRNAs and other miRNAs whose targets are related with metabolism deregulations observed in RCC.

## Data Availability

Data available on request due to ethical restrictions.
